# Diversification of CD1 Molecules Shapes Lipid Antigen Selectivity

**DOI:** 10.1093/molbev/msab022

**Published:** 2021-02-02

**Authors:** Nicole M Paterson, Hussein Al-Zubieri, Matthew F Barber

**Affiliations:** 1 Institute of Ecology and Evolution, University of Oregon, Eugene, OR 97403, USA; 2 Department of Chemistry and Biochemistry, University of Oregon, Eugene, OR 97403, USA; 3 Department of Biology, University of Oregon, Eugene, OR 97403, USA

**Keywords:** adaptive immunity, positive selection, antigen presentation, protein evolution

## Abstract

Molecular studies of host–pathogen evolution have largely focused on the consequences of variation at protein–protein interaction surfaces. The potential for other microbe-associated macromolecules to promote arms race dynamics with host factors remains unclear. The cluster of differentiation 1 (CD1) family of vertebrate cell surface receptors plays a crucial role in adaptive immunity through binding and presentation of lipid antigens to T-cells. Although CD1 proteins present a variety of endogenous and microbial lipids to various T-cell types, they are less diverse within vertebrate populations than the related major histocompatibility complex (MHC) molecules. We discovered that CD1 genes exhibit a high level of divergence between simian primate species, altering predicted lipid-binding properties and T-cell receptor interactions. These findings suggest that lipid–protein conflicts have shaped CD1 genetic variation during primate evolution. Consistent with this hypothesis, multiple primate CD1 family proteins exhibit signatures of repeated positive selection at surfaces impacting antigen presentation, binding pocket morphology, and T-cell receptor accessibility. Using a molecular modeling approach, we observe that interspecies variation as well as single mutations at rapidly-evolving sites in CD1a drastically alter predicted lipid binding and structural features of the T-cell recognition surface. We further show that alterations in both endogenous and microbial lipid-binding affinities influence the ability of CD1a to undergo antigen swapping required for T-cell activation. Together these findings establish lipid–protein interactions as a critical force of host–pathogen conflict and inform potential strategies for lipid-based vaccine development.

## Introduction

Early detection of pathogen-specific molecules by the immune system can mean the difference between resistance, latency, or succumbing to infectious disease. Previous studies have illustrated that host–pathogen protein interaction surfaces are hotspots for repeated natural selection by influencing resistance or susceptibility to infection (Daugherty and Malik 2012; Enard et al. 2016; van der Lee et al. 2017). Such conflicts between hosts and pathogens can give rise to a variety of evolutionary dynamics including Red Queen arms races (Van Valen 1973; Daugherty and Malik 2012), frequency-dependent selection (Takahata and Nei 1990), and over-dominance (Hughes and Nei 1990; Takahata and Nei 1990; Nei and Rooney 2005). Although vertebrate immune systems are tuned to recognize a wide variety of pathogen-associated macromolecules including DNA, RNA, lipids, and glycans, our understanding of host–pathogen evolutionary conflicts is largely restricted to protein–protein interactions (Sawyer et al.2005; Elde et al. 2009; Barber and Elde 2014; Choby et al. 2018). In the case of lipid and lipopeptide antigens, the production of a functional molecule involves the synthesis of precursors that are further processed by enzymatic modifications. As such, evolutionary dynamics involving these macromolecules and their host receptors may be distinct from protein–protein interactions.

The major histocompatibility complex (MHC) superfamily comprises a variety of cell surface proteins which present self and foreign antigens to T-cells. Recognition of foreign antigens by T-cell receptors (TCRs) leads to T-cell activation and initiation of an adaptive immune response (Frank 2002). Multiple evolutionary forces are hypothesized to contribute to the immense diversity in MHC haplotypes, including overdominance wherein increased heterozygosity is favored by selection and polymorphisms are maintained over time (Takahata and Nei 1990). In addition to class I and class II MHC molecules which present peptide antigens, the paralogous cluster of differentiation 1 (CD1) and MR1 molecules have been shown to present lipid and lipoprotein antigens to T-cells (Blumberg et al. 1995; Barral and Brenner 2007; Birkinshaw et al. 2015; Moriet al.2016; Zajonc and Flajnik 2016). CD1 molecules display rare and infrequent polymorphism with limited genetic diversity within humans and other populations relative to class I and II MHC (Han et al. 1999; Golmogghaddam et al. 2013). The MHC and CD1 gene families therefore appear to have experienced divergent evolutionary paths after their duplication from a common ancestor with respect to antigen recognition and population genetic variation. CD1 paralogs are divided into groups, whereby group 1 CD1 family members (including human CD1a, CD1b, and CD1c) present antigen primarily to cytotoxic CD8+ T-cells (Mori et al.2016). Group 2 CD1 molecules (including CD1d in humans) present antigens to invariant natural killer (iNK) T-cells (Pereira and Macedo 2016).

**Fig. 1.**
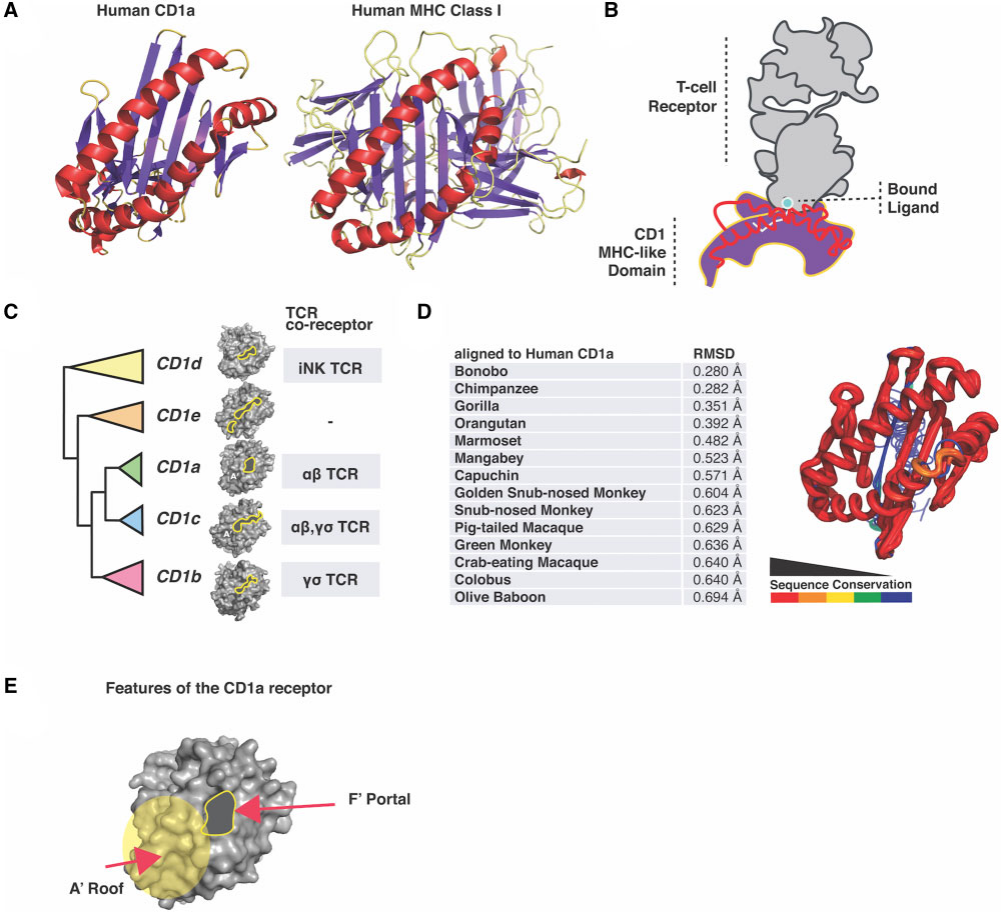
Diversity of the CD1 gene family in primates. (*A*) Ribbon diagrams of human CD1a (PDB ID: 5J1A) and MHC (PDB ID: 1AKJ) with alpha helices highlighted in red, beta-sheet in purple, loops in yellow. (*B*) Illustration of the CD1-TCR interaction where CD1 bind lipid tails in a hydrophobic pocket with polar head groups typically exposed. The TCR (gray) “reads” the displayed antigen leading to T-cell activation. (*C*) Cladogram representing phylogenetic relationship of primate CD1a-e paralogs used in this study with surfaces generated in PyMol and antigen-binding pockets outlined in yellow (PDB IDs: 5J1A, 4ONO, 4MQ7, 3S6C). TCR types that recognize each CD1 paralog are also indicated. (*D*) Primate CD1a diverges most in the lipid-binding domain, which may alter pocket morphology and TCR interactions. Most of the sequence divergence in the primate CD1a proteins is predicted to exist in the beta-sheet that transects the center of the protein with some variation in the central surface region. RMSD, root mean square deviation. (*E*) Structural features of the CD1a receptor. Labels showing location of A′ Roof, F′ portal.

CD1 and MHC (fig. 1*A*) arose in jawed fishes with the advent of the B-cell and T-cell immune receptors during the genesis of the adaptive immune system of vertebrates (Barral and Brenner 2007). After the gene duplications that gave rise to ancestral MHC and CD1, vertebrate CD1 paralogs expanded through repeated duplication events (Dascher 2007). This initial expansion was followed by differential pseudogenization and expansion of CD1 paralogs to various degrees across vertebrate species (Rogers and Kaufman 2016). As such, CD1 gene content varies widely across vertebrates: primates have a single copy of the CD1A paralog, mice possess none, dogs encode six, and horses possess five (Dascher 2007). In primates, CD1D is believed to represent the most deeply conserved member of the CD1 family (Salomonsen et al.2005). CD1d receptors can display antigen to specialized iNKT-cells, which are able to mount an earlier response to infection reflecting their dual role in innate and adaptive immunity (Pereira and Macedo 2016). Current evidence indicates that human CD1e does not present antigen (Garcia-Alles et al. 2011) but rather assists in antigen loading onto CD1d in lysosomes and endosomes (Cala-De Paepe et al. 2012). This wide range of responsive T-cell types along with evidence that these nonclassical T-cell types mount an early immune response to infection (Godfrey et al. 2015) makes human CD1-expressing cells surprisingly flexible responders to infections even with their lack of exceptional sequence variation.

CD1 molecules possess an extracellular domain containing a subsurface hydrophobic-binding pocket used to present antigen to CD1-restricted T-cells (fig. 1*B*) (Barral and Brenner 2007). During the adaptive immune response, CD1 on the surface of antigen-presenting cells activates T-cells by displaying specific classes of hydrophobic ligands to TCRs (fig. 1*C*) (Blumberg et al. 1995; Barral and Brenner 2007; Chancellor et al.2018). According to structural studies, CD1a has the smallest of the human CD1-binding pockets with a volume of about 1,280 Å (Ly and Moody 2014). After the gene duplication event that gave rise to this paralog, CD1a likely evolved to present either self-lipids or small exogenous lipopeptides (Mori et al.2016). Consistent with this hypothesis, CD1a has been crystallized in complex with self-lipids sphingomyelin, lysophosphatidylcholine (Birkinshaw et al. 2015), sulfatide (Zajonc et al. 2003), as well as the mycobacterial lipopeptideanalog didehydroxy-mycobactin (Zajonc et al. 2005). The binding pocket of human CD1a is composed of a double-chambered cavity termed the A′ and F′ pockets with a single (A′) portal that coordinates the presentation of lipid antigen to the TCR (fig. 1*B*) (Zajonc et al. 2005). The TCR lands just above the A′ pocket on a surface termed the A′ roof (Zajonc et al. 2005). The diminutive size of the CD1a A′ pocket is thought to be formed by the electrostatic interaction of the two side chains belonging to the A′ roof that also draw the two parallel alpha helices of the pocket in close proximity, whereas an amino acid sidechain blocks the base of the pocket thereby limiting size of tail groups that can be accommodated (in human CD1a this amino acid is valine 28) (Zajonc et al. 2003). Several other CD1 homologs, except for CD1c, lack this roof structure (Blumberg et al. 1995). CD1a does not feature a late endosomal targeting element and does not require low pH for antigen binding as is the case for other CD1 proteins such as CD1b, CD1c, and CD1d (Chancelloret al.2018).

The immense diversity of the MHC family within and between populations at surfaces necessary for peptide antigen recognition has made these genes classic study systems of adaptive protein evolution (Danchin and Pontarotti 2004; Castroet al. 2015; Grimholt 2016). CD1 molecules possess similar structure and function to class I and II MHC proteins, although their relative lack of diversity at the population level has been attributed to a lack of diversity in their cognate lipid ligands. Although variation in pathogen-derived lipids has been implicated in host immune recognition and virulence (Chandler et al. 2020), the potential for lipid antigens to promote evolutionary conflicts with host species is unclear. In the present study, we used the CD1 family as a system to investigate the diversity and evolution of lipid antigen recognition by the vertebrate immune system.

## Results

### Diversification of the CD1 Gene Family in Primates

A comparison of MHC class I and CD1 protein structures illustrates the homology between these antigen presentation molecules (fig. 1*A*). CD1 presents antigen to the TCR with the lipid tail groups tucked into the hydrophobic pocket and head groups exposed where they are “read” by the TCR (fig. 1*B*). Distinct CD1 molecules present antigen to a wide variety of T-cell types (fig. 1*C*) (Godfrey et al. 2015). To assess patterns of genetic diversity among primate CD1 family members, we first assembled a collection of simian primate CD1 homologs from publicly available genome databases and generated a phylogenetic gene tree using PhyML (fig. 1*C* and supplementary fig. S1, Supplementary Material online). The five human CD1 paralogs are present in the majority of primate genomes surveyed, allowing us to reliably compare structural and genetic diversity within this family. A comparison of the sequences between CD1 orthologs revealed a striking degree of diversification, particularly in the MHC-like domain responsible for lipid antigen presentation (fig. 1*D*). To assess the potential consequences of this variation on CD1 function, we plotted the structural conservation among primate CD1a orthologs using color by conservation (Mura et al. 2010) (fig. 1*D* and *E*). Our analysis revealed several hotspots of high amino acid divergence among CD1 molecules, focused on both interior regions of the antigen-binding pocket as well as surface helices that are known to contact the TCR. Together our results indicate that, despite their limited polymorphism within populations, CD1 paralogs exhibit a high degree of genetic divergence between simian primate species.

### Signatures of Repeated Positive Selection Acting on Primate CD1 Genes

**Fig. 2.**
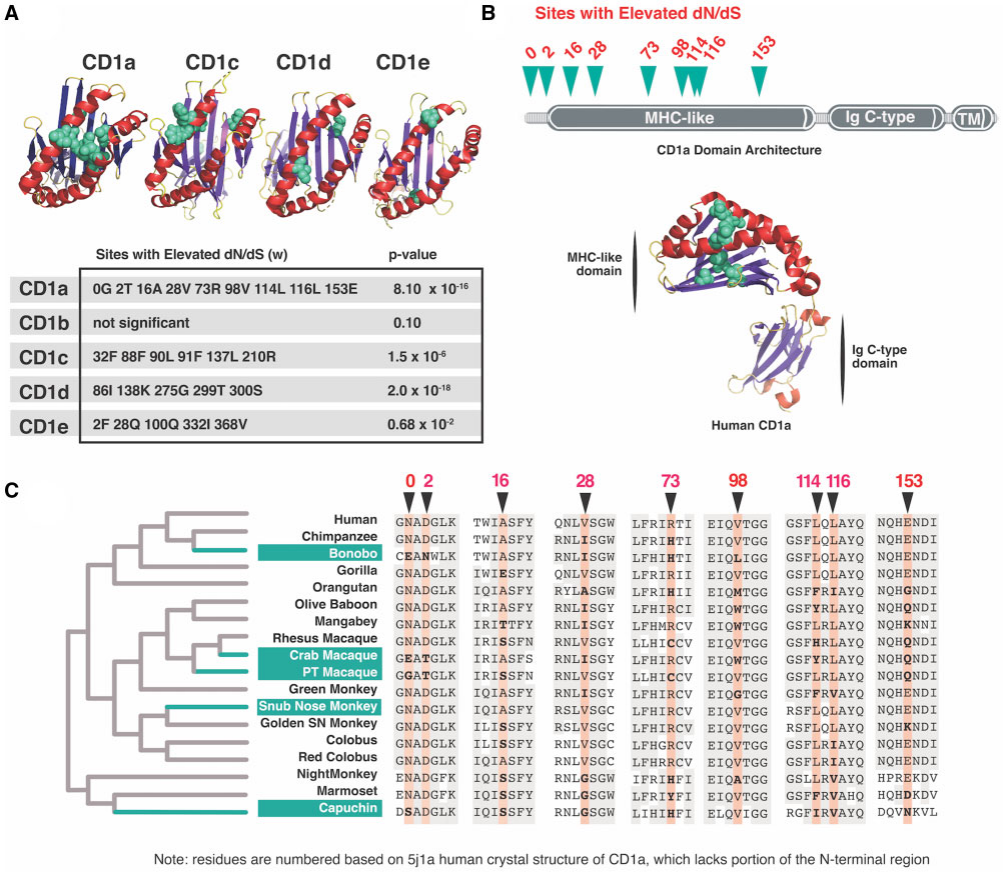
Evidence of repeated positive selection among primate CD1 orthologs. (*A*) Amino acid sites exhibiting strong signatures of positive selection (elevated d*N*/d*S*) are highlighted in teal and mapped onto corresponding crystal structures. Alpha helices are denoted in red, beta-sheet in purple. (PDB IDs: 5J1A, 4ONO, 4MQ7, 3S6C). Table summarizes positions in CD1 paralogs contributing to signatures of positive selection as well as statistics from PAML M7-M8 model comparisons. (*B*) Sites with elevated d*N*/d*S* indicative of positive selection (teal) cluster in the MHC domain of CD1a protein (PDB ID: 5J1A). Alpha helices denoted in red, beta-sheet in purple. (*C*) Multiple sequence alignment of primate species used to calculate d*N*/d*S* ratios for CD1a paired with phylogenetic species tree highlighting the branches (teal) predicted by aBSREL to be undergoing episodic positive selection.

Given their relative lack of within-population diversity, we were surprised by the elevated genetic divergence between primate CD1 orthologs. Previous studies of CD1A genetic diversity in humans revealed only three low-frequency polymorphisms (Han et al. 1999). Across primates included in this study, however, only 52.8% sites are identical. We hypothesized that this variation could be the result of repeated positive selection in response to diverse lipid antigen structures. To identify potential amino acid sites that may have been subject to repeated positive selection, we used the codeml package from PAML (Yang 2007) in addition to MEME (Murrell et al. 2012) and FuBar (Murrell et al. 2013) algorithms from the HyPhy software package to detect elevated d*N*/d*S* (ω) at sites in CD1 paralogs across primates. Elevated ω values were detected for all CD1 family members (fig. 2*A*) with the exception of CD1b, consistent with elevated nonsynonymous substitution rates associated with positive selection. We noted that the majority of rapidly-evolving sites among CD1 paralogs were focused in the MHC-like domain which is responsible for lipid binding (fig. 2*A*). These results suggest that multiple members of the CD1 family have undergone repeated episodes of positive selection in simian primates specifically within regions important for lipid antigen presentation.

Having detected evidence of positive selection acting on several CD1 family members, we chose CD1a for additional in-depth analysis. CD1a has less stringent lipid loading requirements than other CD1 homologs as it does not require a reduced pH environment encountered in late endosomes, nor does it have a known adapter protein required for antigen loading (Barral and Brenner 2007). For these reasons, we anticipated that empirical and molecular modeling studies of antigen recognition would be less complex for CD1a than other paralogs. CD1a has been shown to present mycobacterial antigens from the cell surface where it interacts with langerin on Langerhans cells (Mizumoto and Takashima 2004), a specialized dendritic cell type that surveys epithelial monolayers for molecular indicators of infection.

**Fig. 3.**
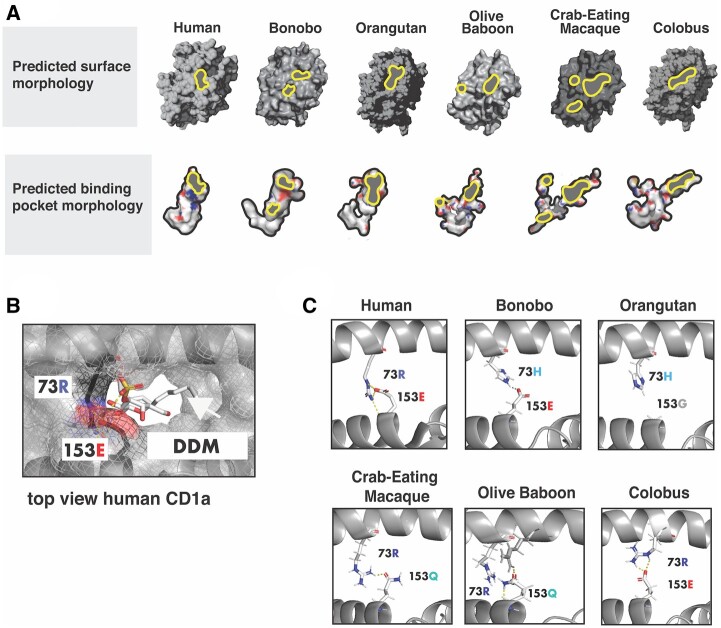
Structural modeling illustrates diversity at the CD1a T-cell interaction interface. (*A*) Predicted attributes of various primate CD1a structures. Surface characteristics across selected primates reveal differences in portal size, number of portals, and pocket morphology. Portals where T-cell receptor “reads” head group are highlighted with gray/yellow outlines. Pocket morphologies and electrostatic properties are shown below surface models. (*B*) PyMol generated top-view of human CD1a bound to dideoxy-mycobactin (PDB ID: 1XZO). Rapidly-evolving positions 73 and 153 coordinate head groups of antigenic ligand. Note hydrogen bonding between head group and 153E. (C) Primate CD1a A′ roof predicted structures where CD1a interacts with TCR. Notably, the orangutan model does not form roof structure due to mutation at site under selection. Olive baboon and crab-eating macaque form A′ roof with residue of differing property at site 153.

To determine what domains of CD1a are subject to positive selection, we mapped the sites with high ω values from our previous analysis. Results show clustering of rapidly evolving sites in the MHC-like domain, similar to those observed with other CD1 paralogs (fig. 2*B* and supplementary figs. S2–S4, Supplementary Material online). These sites also map to regions where CD1a is likely to interface with lipid antigen or the TCR, suggesting that selection may have acted to alter lipid-binding and T-cell interactions. The majority of variable sites between CD1a orthologs cluster to a region of the protein near the center of the binding pocket and around the outer surface (fig. 2*B*). We grouped all of the rapidly-evolving sites we identified in this study into three categories: residues located at or near the TCR landing site (the A′ roof in the human structure), residues within the binding pocket, and residues in the N-terminus for which we have no structural information. Overall, predicted structural features do not correlate well with phylogenetic relatedness, consistent with multiple lineages undergoing episodic selection (figs. 2*C* and 3*A*).

### Accelerated Evolution of the CD1a-TCR Interface

To assess how variation in CD1a may influence immune functions in primates, we used I-TASSER to generate predicted structures of several CD1a orthologs (fig. 3*A*). Of the hominoid structures modeled, human, bonobo, and orangutan have remarkably different topologies at the TCR-CD1a interface as well as the geometry of the internal binding pocket (fig. 3*A*). The morphologies of the binding pockets vary widely, most notably in the crab-eating macaque which is predicted to contain one main and two accessory portals, with a narrow meandering channel (fig. 3*A*, bottom panel). The length and volume of the pockets limit the types of lipid tail groups that can be accommodated, whereas the size and location of the portals have effects on how well the T-cell receptor can read the antigen presented (Birkinshaw et al. 2015).

In human CD1a, the A′ roof is hypothesized to aid in determining whether an antigen will elicit an immune response by supporting interactions with the TCR and assisting in display of the ligand head-group (fig. 3*B*) (Zajonc et al. 2005; Birkinshaw et al. 2015). The predicted orangutan CD1a structure lacks an A′ roof entirely (fig. 3*C*), whereas bonobo CD1a possesses two portals. Additionally, it has been speculated that disruption of hydrogen bonding between R73, R76, and E154 that form the A′ roof may indicate whether a given ligand will stimulate TCR activation (Birkinshaw et al. 2015). However, several of the CD1a structures are predicted to form an A′ roof that does not depend on this particular interaction. For example, crab-eating macaque and olive baboon CD1a are predicted to form a relatively unique A′ roof composed of an R73/153Q linkage that does not involve R76 (fig. 3*C*). The TCR does not recognize CD1a-bound ligands without adequate projection of lipid head groups, and it is likely that hydrogen bonding between the head groups of smaller ligands and residues that make up the portal are important for display. Headless ligands buried in the CD1a pocket, for example, can result in T-cell auto-reactivity (de Jong et al. 2010). Site 153, which is highly variable across primates (fig. 2*D*) has been shown to form a hydrogen bond in human CD1a to the head group of self antigen lysophosphatidylcholine and sulfatide in addition to its role in forming the A′ roof (Zajonc et al. 2003; Birkinshaw et al. 2015). This site bears a glycine in orangutan, with no ability to form an A′ roof or salt bridges with ligand (fig. 3*C*). Together, these predicted structural differences suggest that natural selection may have had a significant impact on the ability of CD1a to display self or foreign lipid antigens across related primates.

### Structural Remodeling of the CD1a Antigen-Binding Pocket

To determine whether the structural differences observed across CD1a paralogs are likely to have functional consequences for antigen recognition, we applied a ligand-docking approach using AutoDock Vina. We were particularly interested to test affinity differences between endogenous and exogenous lipid ligands, since the current model for CD1a antigen presentation is a swapping mechanism wherein lower affinity endogenous ligand is replaced at the cell surface by higher affinity exogenous ligand. We used ligands previously crystallized in complex with human CD1a in our studies since there is a wealth of structural information available on these particular binding interactions. In our docking simulations, we found that a single loop region is required to redock all ligands in the CD1a-binding pocket. We assigned flexibility to this region for all the structures tested, as well as any nonbonded sidechains in the region of the portal (supplementary fig.S2, Supplementary Material online). We believe this is likely the region responsible for conferring flexibility in the native CD1a, which must be flexible enough to accommodate ligands with a diversity of molecular weights (molecular weight of urushiol is 330 g/mol, dideoxy-mycobactin is 838 g/mol).

**Fig. 4.**
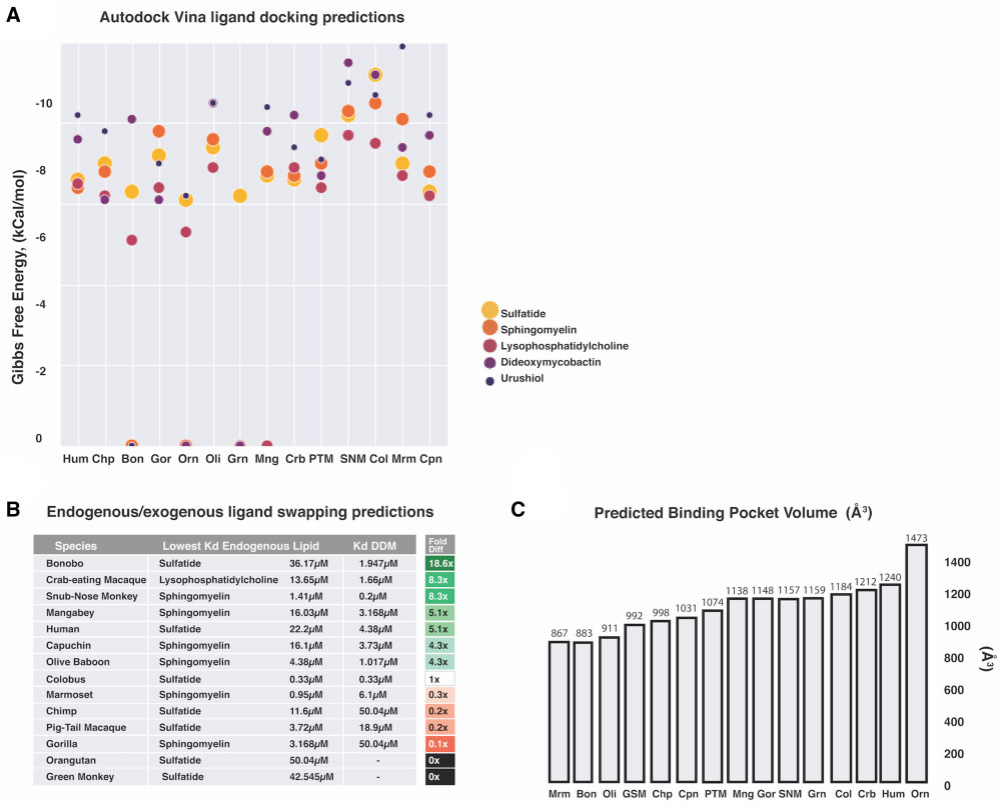
Divergence of CD1a shapes predicted endogenous and exogenous lipid antigen affinities. (*A*) Plot of relative Gibbs free energy values for all ligands tested by ligand docking predictions using AutoDock Vina. Lowest energy values for each set are plotted. Sulfatide, sphingomyelin, lysophosphatidylcholine are endogenous lipid ligands. Dideoxymycobactin (DDM) is a synthetic lipid analog of *Mycobacterium tuberculosis* siderophore mycobactin. Urushiol is the etiological agent of poison ivy rash. (*B*) Lipid-swapping predictions based on predicted Kd (dissociation constant) from docking studies. (C) Predicted pocket volume for CD1a orthologs. Legend: Hum, Human; Chp, Chimpanzee; Bon, Bonobo; Gor, Gorilla; Orn, Orangutan; Oli, Olive Baboon; Grn, Green Monkey; Mng, Mangabey; Crb, Crab-eating macaque; SNM, Snub-nosed monkey; Col, Colobus;Mrm, Marmoset;Cpn, Capuchin.

We next measured predicted CD1a-binding affinities for the panel of lipid ligands including endogenous ligands sphingomyelin, sulfatide, lysophosphatidylcholine, and exogenous ligand dideoxy-mycobactin (DDM) (fig. 4*A* and supplementary figs. S5–S7, Supplementary Material online). We chose these lipids because published structural information exists for each ligand bound to the human CD1a receptor. Given that CD1a is believed to swap endogenous lipids for exogenous lipids based on differences in relative binding affinities, we then estimated the likelihood of a lipid-swap using our panel of ligands. We calculated the fold differences in Kd (dissociation constant) between the highest affinity endogenous lipid and compared this to the Kd of the exogenous ligands. Crab-eating macaque, snub-nosed monkey, olive baboon, capuchin, mangabey, bonobo, and human were predicted to swap out endogenous for DDM (fig. 4*B*). It is worth noting in this case that we assume the endogenous lipid with the lowest Kd is also present in abundance, which we can not know for certain *in vivo*. It has been shown in previous studies that human CD1a molecules bind to a diverse repertoire of lipid types *in vitro* (Birkinshaw et al. 2015). Since lipid profiles are not available for all cell types in the primates we studied, we chose this simplification as a rough estimate for the feasibility of lipid swapping.

A notable result from these ligand docking predictions was that binding profiles failed to group by species phylogeny, consistent with branch-site test results that detected several branches undergoing multiple bouts of episodic positive selection (supplementary fig.S5, Supplementary Material online). Unlike in humans where the largest binding pocket (CD1b) (Ly and Moody 2014) also has the most promiscuous ligand-binding profile, ligand docking predictions do not group higher affinity binding with predicted pocket volume (fig. 4*C* and supplementary fig.S8, Supplementary Material online). These findings indicate that predicted structural alterations in the CD1a ligand-binding pocket have significant impacts on recognition of both endogenous and pathogen-derived antigens which collectively shape downstream T-cell activation.

### Modeling the Effects of Rapidly-Evolving Sites on Antigen Presentation by CD1a

**Fig. 5.**
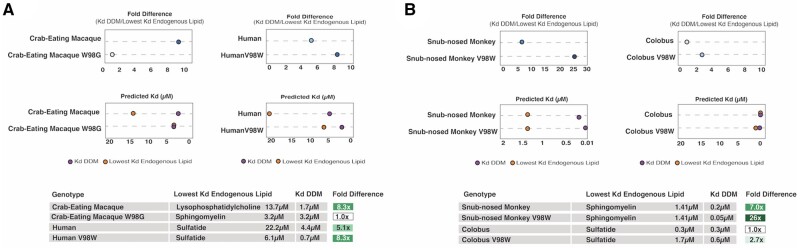
Rapidly-evolving positions in CD1a are sufficient to modulate predicted affinity for lipid antigens. (*A*) Mutation of site 98 to tryptophan in human CD1a (olive baboon and crab-eating macaque share this amino acid at this position) results in increased predicted binding affinity to DDM, with overall fold increase between endogenous ligand and DDM. Mutation of tryptophan at site 98 in crab-eating macaque to ancestral glycine results in higher binding affinity for all endogenous ligands tested, and loss of feasible lipid-swapping and DDM presentation. (*B*) Mutation of site 98 to tryptophan in snub-nosed monkey CD1a results in increased predicted binding affinity to DDM. Colobus, which is not predicted to swap endogenous ligand for DDM, also increases spread between binding affinities. In colobus, however, it is a decrease in affinity for endogenous lipid rather than increase in DDM affinity that is responsible for the fold change.

**Fig. 6.**
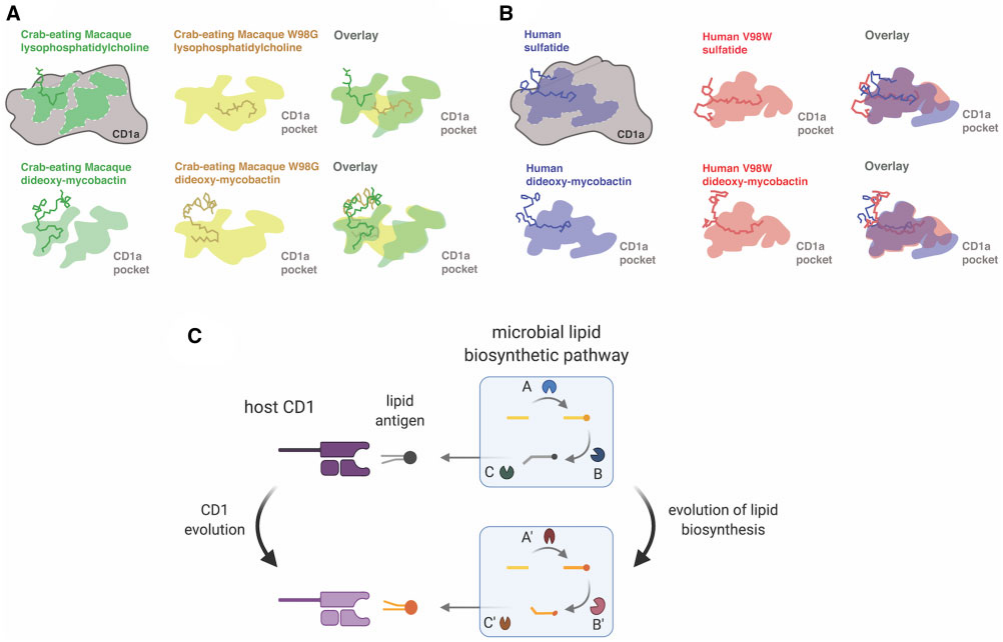
Conceptual framework for lipid-driven diversification of CD1 molecules. (*A*) Crab-eating macaque CD1a, which encodes a tryptophan in position 98, is predicted to lose the ability to present self-lipid lysophosphatidylcholine when this position is mutated to the consensus at this site, glycine. An overlay of the differences in pocket morphology shows how the tryptophan limits access to the deeper chambers of the pocket. (*B*) Humans possess a valine at position 98, which has been proposed to act as a barrier limiting larger ligands access to the pocket. When this residue is mutated to a tryptophan *in silico*, further decreasing access to the deeper chambers of the pocket, the ability to swap out endogenous for exogenous ligand is improved, suggesting that a large hydrophobic residue in this position may be beneficial in the context of *Mycobacterium tuberculosis* infection in primates. Cartoons were informed by analysis of Autodock Vina docking results analyzed in PyMol. (*C*) Conceptual framework for lipid-driven evolution of CD1, resulting in accelerated evolution and rapid diversification of host immune receptors. Lipid biosynthesis pathways are complex and interdependent, thereby adding levels of complexity that may slow the rate at which pathogens can successfully evolve new lipid antigens. Figure created using Biorender.com.

We next assessed how variation at single rapidly-evolving positions in the CD1a-binding pocket may alter lipid antigen recognition. Using PyMol, we substituted single extant amino acids for the ancestral amino acid at sites undergoing positive selection (ancestral sites predicted by DataMonkey package SLAC; Pond and Frost 2005) and used these altered structures in our ligand-docking simulation. We then tested the effects of mutations in positively-selected sites on crab-eating macaque CD1a. We observed that the W98G substitution (which replaces a bulky tryptophan at the base of the pocket for the smallest residue, ancestral glycine) significantly increasedbinding affinity for endogenous lipids in crab-eating macaque, thus making it unlikely that swapping for DDM would occur (fig. 5*A*). This mutation appears to have similar effects in other genetic backgrounds as well, including humans (fig. 5*A* and *B*). Analysis of the binding pose in crab macaque W98G bound to lysophosphatidylcholine shows the ligand buried in the pocket without an exposed head group (de Jong et al. 2010) (fig. 6*A*). This provides a possible explanation for why the reduction in accessible pocket volume may be beneficial, both for lipid swapping and TCR ligand recognition. In the human V98W mutation, we noticed that the tail group accesses deeper regions of the pocket, which may partially explain the higher affinity for DDM seen in this model (fig. 6*B*).

To probe our system further, we used the genetic background of snub-nosed monkey to simulate the effects of mutations since it encodes primate consensus residues at positions with elevated d*N*/d*S*. We mutated seven sites that appear at the interaction interface to the ancestral sites at all loci, resulting in a protein that is not likely to swap endogenous ligand for DDM by our predictions (supplementary fig.S6, Supplementary Material online). Smaller effect mutations were identified when introducing combinations of mutations in crab-eating macaque at position 114 where tyrosine appears to lower affinity for endogenous ligand and increases affinity for mycobacterial ligand slightly (supplementary fig.S7, Supplementary Material online). Taken together, several species are predicted to bind DDM with relatively high affinity but may not necessarily present exogenous antigen due to equally or greater affinity for endogenous lipid. This suggests that selective pressure may exist to decrease affinity for endogenous ligand in conjunction with increased affinity for exogenous antigens, resulting in increased effectiveness of CD1a-dependent immune responses. We observed that even single substitutions in rapidly-evolving sites substantially alter both endogenous and pathogen-derived lipid antigen recognition, providing further evidence for the functional impact of divergence in CD1a.

## Discussion

Overdominance has been proposed as an important force acting on MHC genes producing diversity across the gene family (Hughes and Nei 1990). CD1 genes exhibit limited sequence variation within humans (Blumberg et al. 1995), which suggests overdominance is likely not a major factor shaping the evolution of this family. Rather, our observations of elevated d*N*/d*S* between CD1 orthologs and limited polymorphism within species are most consistent with a history of repeated selective sweeps driven by positive selection. Moreover, the patterns of divergence in CD1, with amino acid variation enriched within the MHC-like domain, supports the hypothesis that lipid antigen recognition and presentation are the functional drivers of this divergence. These patterns are also observed in MHC genes (which are also undergoing positive selection), with elevated ω at hotspots in the MHC antigen recognition groove (Hughes and Nei 1990; Manlik et al. 2019). The electrostatic property variation in lipid ligands is found almost exclusively in the head-groups, with differences in the tail groups restricted to length and geometry of the hydrocarbon tails. As these tail groups have the most physical contact with CD1-binding pockets, amino acids changes affecting the length and geometry of this pocket determine which hydrophobic chains can be accommodated. Patterns of evolution observed in CD1 could reflect a classical arms race in which host receptors and a subset of microbial antigens antagonistically coevolve through time. Alternatively, selection in a fluctuating environment where the fitness benefit of recognizing a particular lipid antigen changes over time could also produce elevated patterns of divergence in CD1. Coevolution between lipid antigens and host proteins would likely involve mutations in microbial genes responsible for lipid processing or modification (fig. 6*C*). Future studies could aid in determining how variation in lipid-modifying genes shapes CD1-dependent immune responses to specific pathogens.

Collectively our results suggest that, for species predicted to undergo lipid swapping of endogenous lipid for mycobactin, natural selection may have acted to decrease affinity for endogenous ligand while increasing affinity for exogenous antigen by CD1a. We observed that a single substitution can significantly alter the predicted effects of ligand-binding affinity, with potential consequences for antigen presentation (fig. 5*A* and *B*). Notably, a major effect mutation identified in species undergoing episodic bouts of selection has the ability to reliably increase affinity for DDM and/or decrease the affinity of endogenous ligands by CD1a (fig. 5). Our analyses also indicate other residues determining binding pocket volume in human CD1a are undergoing repeated positive selection across primates (fig. 2). In particular, valine 28 has been reported to form a molecular barrier that acts as a size-limiting determinant for antigen binding (Zajonc et al. 2003). Notably, New World monkeys encode a smaller residue (glycine) at this position. Replacement of valine with glycine might be expected to expand the size of the binding pocket. However, our molecular modeling indicates that the binding pocket in the New World monkey lineages is predicted to be smaller than even the crab-eating macaque or mangabey, which bear an isoleucine and a threonine, respectively, at this same site. These observations suggest that molecular determinants of binding pocket volume and morphology are complex and influenced by a combination of variable amino acid substitutions. Additionally, the size of the binding pocket does not appear to correlate with feasibility of DDM presentation. This is notable because in other CD1 molecules multiple lipids can be accommodated, negating the need for a stronger binding affinity for exogenous ligand. In fact, other CD1 molecules such as CD1b may even require “spacer” lipids (Garcia-Alles et al. 2011). These observations may reflect selection acting to produce a binding pocket that is able to swap out endogenous ligand without the need for a loading protein as seen in other CD1 paralogs. This feature enables CD1a to directly surveil the environment for pathogen-associated molecules, a potential advantage compared with the other CD1 molecules which require lysosomal processing and accessory protein loading before antigen presentation can occur at the cell surface.

In order for a microbial pathogen to evolve alternative lipid antigen structures, mutations likely occur in genes responsible for synthesis or modification of the lipid antigen. Mutations in processing and production of lipids will most likely have effects on steps of the biosynthesis pathways that are downstream of the mutated enzyme (fig. 6*C*). In the future, it would be intriguing to test whether primate CD1a orthologs have evolved to detect other lipid types or variations of mycobactin derived from other pathogen sources. According to data from NIHTPR’s AceView (Thierry-Mieg and Thierry-Mieg 2006), gene expression of CD1a/c is exceptionally high in tissues in pig-tailed macaque. Additionally, certain orthologs such as the marmoset CD1a exhibit very low gene expression (Thierry-Mieg and Thierry-Mieg 2006) and may be undergoing rapid birth-and-death evolution (Nei and Rooney 2005) and eventual pseudogenization. Such observations would be consistent with findings of dynamic CD1 gene duplication and loss across vertebrates (Nei and Rooney 2005). The significance of changes in endogenous lipid presentation will also be an area for important future investigation. Certain isoforms of sulfatide, for example, are associated with cancerous cells and when bound to CD1a can prime T-cells(Takahashi and Suzuki 2012). It has also been shown that presentation of endogenous ceramides by CD1d is associated with the ability to detect disease (Paget et al. 2019).

CD1 molecules possess the ability to bind and present hydrophobic antigens from a variety of pathogens, many of which likely remain to be described. It is notable, however, that the majority of CD1 antigens identified to date are derived from pathogenic mycobacteria including *Mycobacterium tuberculosis*, the causative agent of tuberculosis in humans. Tuberculosis remains a devastating human public health burden, recently accounting for more deaths due to infectious disease than any other single pathogen (Forrellad et al. 2013). It is tempting to speculate whether mycobacterial antigens have indeed imposed particularly strong selective pressure on CD1 molecules during animal evolution. Given the limited effectiveness of the current tuberculosis vaccine (Schito et al. 2015; Gonget al.2018), addition of CD1-targeted antigens in a next-generation vaccine could provide one avenue for increased efficacy (Gonget al.2018). Functional characterization of diverse CD1 orthologs beyond humans may reveal whether detection of mycobacterial antigens is a widely conserved feature in this family, as well as possible routes to enhance CD1-mediated immunity against *M. tuberculosis*. Alternatively, evolution-guided development of synthetic lipid antigens that confer increased activation of CD1-responsive T-cells could provide an alternative strategy to enhance lipid-based vaccines.

Although we focused our molecular modeling and simulation studies on CD1a, comparable signatures of positive selection were identified in primate CD1c, CD1d, and CD1e. Further investigation of these receptors and their cognate antigens would greatly advance our understanding of the importance for CD1 diversity in the evolution of vertebrate immunity. For this study, Autodock Vina was used because published results show strong correlation between docking and experimental values (Trott and Olson 2009) especially when iterations are increased (Jaghoori et al. 2016) as we did in this study. . Additionally, Autodock Vina has been reported to perform well with lipid ligands specifically (Gathiaka et al. 2013). However, there is improved reliability when comparing docking results from the same receptor molecule bound to variable ligands (Jaghoori et al. 2016). The main caveats of this analysis exist in the uncertainties inherent in the structural prediction models. I-TASSER predictions are often very good, but rely on availability of structural information on similar molecules in the database which may not be available (Yang and Zhang 2015).

Although lipids and other pathogen-derived macromolecules have long been appreciated as critical targets for host innate and adaptive immune responses, the potential for these factors to promote evolutionary conflicts with host species has been relatively unexplored. By combining comparative genetics and molecular modeling approaches, this study illuminates how lipid antigens have shaped fundamental features of primate immunity and the detection of globally devastating pathogens.

## Materials and Methods

### Phylogenetic Analyses

A gene tree of primate CD1 was generated with PhyML (phylogenetics by maximum likelihood) with Bayes selection criterion and 1,000 bootstraps (Yang 2007). Between 18–21 primate cDNA sequences were aligned for each CD1A-E gene using MUSCLE (supplementary fig. S8, Supplementary Material online), sequences were trimmed manually using the species phylogeny as reported by Perelman et al.(2011). Our CD1A data set included all available nucleotide coding sequences (cDNA) for 19 primate species, with areas of ambiguity and stop codons removed. Positively selected sites for all CD1 genes were detected using the phylogenetic analysis by maximum likelihood (PAML) software package with F3X4 codon frequency model. Likelihood ratio tests compared pairs of site-specific models M1 with M2 (neutral and selection, respectively), M7 with M8 (neutral, beta distribution of d*N*/d*S* < 1; selection, beta distribution d*N*/d*S* > 1, respectively). Additional tests were performed which account for synonymous rate variation and recombination, including FuBAR (Murrell et al. 2013) and MEME (Murrell et al. 2012), using the HyPhy software package (Murrell et al. 2012, 2013). We chose a stringent selection criteria for the sites we focused on in this study: PAML and FuBAR posterior probability of greater than or equivalent to 0.9, MEME *P* value of 0.1 or less. All sites analyzed (unless otherwise stated) fit these criteria under all three tests.

### CD1a Structural Predictions

The Eukaryotic Linear Motif resource (Kumar et al. 2019) (http://elm.eu.org/, last accessed February 12, 2021) was used to identify structural motifs from the primary amino acid sequence of CD1a. Primate CD1a structures were predicted with amino acid sequences submitted to I-TASSER server (Yang and Zhang 2015) (https://zhanglab.ccmb.med.umich.edu/, last accessed February 12, 2021) to generate structures for analysis using PyMol, primate structural alignment from 14 primate structures colored by conservation based on RMSD calculations from PyMol alignment (https://pymolwiki.org/index.php/Color_by_conservation, last accessed February 12, 2021) (Mura et al. 2010), CASTp for volume predictions, and for use in ligand docking simulations. To assess confidence in our structural predictions, isoform 1 of full-length human CD1a was analyzed (there are several crystal structures available for this molecule) with a *C*-score of −0.36. *C*-score values vary from −5, 2 with positive values indicating higher confidence, and only structures with *C* values between −1 and 2 were used for analysis. Structures were analyzed using PyMol (The PyMol Molecular Graphics System, Version 2.0 Schrödinger, LLC). For binding pocket volume predictions, CASTp was used and the radius probe was set at 0.75 Å for each iTASSER-predicted structure submitted for analysis. The predicted volumes for all species were plotted using the Seaborn package in Python.

### Ligand Docking with AutoDock Vina

Redocking with human CD1a was first performed to identify flexible residues required for all known ligands to redock in the same model. We calibrated our modeling by redocking known ligands in our human CD1a iTASSER-predicted structure. According to our calculations, a comparison of CD1a crystal structures bound to the smallest and largest ligands (PDB ID 4X6D, 1XZ0) yields an RMSD of 1.23 Å. This suggests there is flexibility in the CD1a pocket, supported by a number of hydrogen bonds between residues of the main CD1a-binding domain alpha helices. Dorsal loop of alpha helix 2 was identified as required and made flexible in all primate CD1a structures analyzed. Receptors with amino acid side chains that occluded the binding pocket were also made flexible if not engaged in hydrogen bonds, and any of these additionally flexible residues (see Supplementary Material online for details). AutoDockTools 1.5.6 (Trott and Olson 2009) was used to prepare the ligands and receptors for ligand docking. AutoDock Vina was run in the command line and docking results were analyzed in PyMol and plotted with the Python Seaborn package. A Python script was written to perform K_D_ calculations. Details of Vina settings including exhaustiveness, grid center, and *x*, *y*, *z* coordinates are available in the Supplementary Material online.

## Data Availability

The data underlying this article are available in the article and in its online supplementary material.

## Supplementary Material

Supplementary data are available at *Molecular Biology and Evolution* online.

## Author Contributions

N.M.P. and M.F.B. conceived the study. N.M.P. performed all phylogenetic analyses, structural modeling, and ligand-binding simulations with assistance from H. A-Z. N.M.P. prepared the original manuscript and figures with assistance from M.F.B. All authors reviewed and edited the manuscript.

## Supplementary Material

msab022_Supplementary_Data

## References

[msab022-B1] Barber MF , Elde NC. 2014. Escape from bacterial iron piracy through rapid evolution of transferrin. Science 346(6215):1362–1366. 25504720 10.1126/science.1259329 PMC4455941

[msab022-B2] Barral DC , Brenner MB. 2007. CD1 antigen presentation: how it works. Nat Rev Immunol. 7(12):929–941. 18037897 10.1038/nri2191

[msab022-B3] Birkinshaw RW , Pellicci DG, Cheng T-Y, Keller AN, Sandoval-Romero M, Gras S, de Jong A, Uldrich AP, Moody DB, Godfrey DI, et al 2015. Αβ T cell antigen receptor recognition of CD1a presenting self lipid ligands. Nat Immunol. 16(3):258–266. 25642819 10.1038/ni.3098 PMC7103088

[msab022-B4] Blumberg RS , Gerdes D, Chott A, Porcelli SA, Balk SP. 1995. Structure and function of the CD1 family of MHC-like cell surface proteins. Immunol Rev. 147(1):5–29. 8847079 10.1111/j.1600-065x.1995.tb00085.x

[msab022-B5] Cala-De Paepe D , Layre E, Giacometti G, Garcia-Alles LF, Mori L, Hanau D, de Libero G, de la Salle H, Puzo G, Gilleron M. 2012. Deciphering the role of CD1e protein in mycobacterial phosphatidyl-myo-inositol mannosides (PIM) processing for presentation by CD1b to T lymphocytes. J Biol Chem. 287(37):31494–31502. 22782895 10.1074/jbc.M112.386300 PMC3438982

[msab022-B6] Castro CC , Luoma AM, Adams EJ. 2015. Coevolution of T-cell receptors with MHC and non-MHC ligands. Immunol Rev. 267(1):30–55. 26284470 10.1111/imr.12327 PMC4544829

[msab022-B7] Chancellor A , Gadola SD, Mansour S. 2018. The versatility of the CD1 lipid antigen presentation pathway. Immunology 154(2):196–203. 29460282 10.1111/imm.12912 PMC5980215

[msab022-B8] Chandler CE , Harberts EM, Pelletier MR, Thaipisuttikul I, Jones JW, Hajjar AM, Sahl JW, Goodlett DR, Pride AC, Rasko DA, et al 2020. Early evolutionary loss of the lipid A modifying enzyme PagP resulting in innate immune evasion in *Yersinia pestis*. Proc Natl Acad Sci USA. 117(37):22984–22991. 32868431 10.1073/pnas.1917504117 PMC7502761

[msab022-B9] Choby JE , Buechi HB, Farrand AJ, Skaar EP, Barber MF. 2018. Molecular basis for the evolution of species-specific hemoglobin capture by *Staphylococcus aureus*. MBio 9(6) e01524–18. 30459189 10.1128/mBio.01524-18 PMC6247092

[msab022-B10] Danchin EGJ , Pontarotti P. 2004. Towards the reconstruction of the bilaterian ancestral pre-MHC region. Trends Genet. 20(12):587–591. 15522451 10.1016/j.tig.2004.09.009

[msab022-B11] Dascher CC. 2007. Evolutionary biology of CD1. Curr Top Microbiol Immunol. 314:3–26. 17593655 10.1007/978-3-540-69511-0_1

[msab022-B12] Daugherty MD , Malik HS. 2012. Rules of engagement: molecular insights from host-virus arms races. Annu Rev Genet. 46(1):677–700. 23145935 10.1146/annurev-genet-110711-155522

[msab022-B13] de Jong A , Peña-Cruz V, Cheng T-Y, Clark RA, Van Rhijn I, Branch Moody D. 2010. CD1a-autoreactive T cells are a normal component of the human Αβ T cell repertoire. Nat Immunol. 11(12):1102–1109. 21037579 10.1038/ni.1956 PMC3131223

[msab022-B14] Elde NC , Child SJ, Geballe AP, Malik HS. 2009. Protein kinase R reveals an evolutionary model for defeating viral mimicry. Nature 457(7228):485–491. 19043403 10.1038/nature07529 PMC2629804

[msab022-B15] Enard D , Cai L, Gwennap C, Petrov DA. 2016. Viruses are a dominant driver of protein adaptation in mammals. ELife 5:e12469. 27187613 10.7554/eLife.12469 PMC4869911

[msab022-B16] Forrellad MA , Klepp LI, Gioffré A, Sabio y García J, Morbidoni HR, Santangelo MDLP, Cataldi AA, Bigi F. 2013. Virulence factors of the *Mycobacterium tuberculosis* complex. Virulence 4(1):3–66. 23076359 10.4161/viru.22329 PMC3544749

[msab022-B17] Frank SA. 2002. Immunology and evolution of infectious disease . Princeton (NJ): Princeton University Press. 20821852

[msab022-B18] Garcia-Alles LF , Giacometti G, Versluis C, Maveyraud L, de Paepe D, Guiard J, Tranier S, Gilleron M, Prandi J, Hanau D, et al 2011. Crystal structure of human CD1e reveals a groove suited for lipid-exchange processes. Proc Natl Acad Sci USA. 108(32):13230–13235. 21788486 10.1073/pnas.1105627108 PMC3156185

[msab022-B61] Gathiaka S, Nanayakkara G, Boncher T, Acevedo O, Wyble J, Patel S, Patel A, Shane ME, Bonkowski B, Wieczorek J, et al. 2013. Design, development and evaluation of novel dual PPARδ/PPARγ agonists. *Bioorg Med Chem Lett.* 23(3):873–879. 10.1016/j.bmcl.2012.11.060 23273519

[msab022-B19] Godfrey DI , Uldrich AP, McCluskey J, Rossjohn J, Branch Moody D. 2015. The burgeoning family of unconventional T cells. Nat Immunol. 16(11):1114–1123. 26482978 10.1038/ni.3298

[msab022-B20] Golmogghaddam H , Arandi Abbas Ghaderi N, Doroudchi M. 2013. Polymorphism in exon 2 of CD1 genes in southwest of Iran. Iran J Public Health. 42(7):775–782. 24427756 PMC3881623

[msab022-B21] Gong W , Liang Y, Wu X. 2018. The current status, challenges, and future developments of new tuberculosis vaccines. Hum Vaccines Immunother. 14(7):1697–1716. 10.1080/21645515.2018.1458806 PMC6067889 29601253

[msab022-B22] Grimholt U. 2016. MHC and evolution in teleosts. Biology 5(1):6. 10.3390/biology5010006 PMC4810163 26797646

[msab022-B23] Han M , Hannick LI, DiBrino M, Robinson MA. 1999. Polymorphism of human CD1 genes. Tissue Antigens 54(2):122–127. 10488738 10.1034/j.1399-0039.1999.540202.x

[msab022-B24] Hughes A , Nei M. 1990. Evolutionary relationships of class II MHC genes in mammals. Mol Biol Evol . 7(December):491–514. 2126590 10.1093/oxfordjournals.molbev.a040622

[msab022-B60] Jaghoori MM, Bleijlevens B, Olabarriaga SD. 2016. 1001 Ways to run AutoDock Vina for virtual screening. *J Comput Aided Mol Des.* 30(3):237–249. 10.1007/s10822-016-9900-9 PMC4801993 26897747

[msab022-B25] Kumar M , Gouw M, Michael S, Sámano-Sánchez H, Pancsa R, Glavina J, Diakogianni A, Valverde JA, Bukirova D, Čalyševa J, et al 2019. ELM – the eukaryotic linear motif resource in 2020. Nucleic Acids Res. 48(D1):D296–306. 10.1093/nar/gkz1030 PMC7145657 31680160

[msab022-B26] Lee R. V D , Wiel L, van Dam TJP, Huynen MA. 2017. Genome-scale detection of positive selection in nine primates predicts human-virus evolutionary conflicts. Nucleic Acids Res. 45(18):10634–10648. 28977405 10.1093/nar/gkx704 PMC5737536

[msab022-B27] Ly D , Moody DB. 2014. The CD1 size problem: lipid antigens, ligands, and scaffolds. Cell Mol Life Sci. 71(16):3069–3079. 24658584 10.1007/s00018-014-1603-6 PMC4160407

[msab022-B28] Manlik O , Krützen M, Kopps AM, Mann J, Bejder L, Allen SJ, Frère C, Connor RC, Sherwin WB. 2019. Is MHC diversity a better marker for conservation than neutral genetic diversity? A case study of two contrasting dolphin populations. Ecol Evol. 9(12):6986–6998. 31380027 10.1002/ece3.5265 PMC6662329

[msab022-B29] Mizumoto N , Takashima A. 2004. CD1a and Langerin: acting as more than Langerhans cell markers. J Clin Invest. 113(5):658–660. 14991060 10.1172/JCI21140 PMC351325

[msab022-B30] Mori L , Lepore M, De Libero G. 2016. The immunology of CD1- and MR1-restricted T cells. Annu Rev Immunol. 34(1):479–510. 26927205 10.1146/annurev-immunol-032414-112008

[msab022-B31] Mura C , McCrimmon CM, Vertrees J, Sawaya MR. 2010. An introduction to biomolecular graphics. PLoS Comput Biol. 6(8):e1000918. 20865174 10.1371/journal.pcbi.1000918 PMC2928806

[msab022-B32] Murrell B , Moola S, Mabona A, Weighill T, Sheward D, Kosakovsky Pond SL, Scheffler K. 2013. FUBAR: a fast, unconstrained Bayesian approximation for inferring selection. Mol Biol Evol. 30(5):1196–1205. 23420840 10.1093/molbev/mst030 PMC3670733

[msab022-B33] Murrell B , Wertheim JO, Moola S, Weighill T, Scheffler K, Kosakovsky Pond SL. 2012. Detecting individual sites subject to episodic diversifying selection. PLoS Genet. 8(7):e1002764. 22807683 10.1371/journal.pgen.1002764 PMC3395634

[msab022-B34] Nei M , Rooney AP. 2005. Concerted and birth-and-death evolution of multigene families. Annu Rev Genet. 39(1):121–152. 16285855 10.1146/annurev.genet.39.073003.112240 PMC1464479

[msab022-B35] Paget C , Deng S, Soulard D, Priestman DA, Speca S, von Gerichten J, Speak AO, Saroha A, Pewzner-Jung Y, Futerman AH, et al 2019. TLR9-mediated dendritic cell activation uncovers mammalian ganglioside species with specific ceramide backbones that activate invariant natural killer T cells. PLoS Biol. 17(3):e3000169. 30822302 10.1371/journal.pbio.3000169 PMC6420026

[msab022-B36] Pereira CS , Macedo MF. 2016. CD1-restricted T cells at the crossroad of innate and adaptive immunity. J Immunol Res. 2016:1–11. 10.1155/2016/2876275 PMC5192300 28070524

[msab022-B37] Perelman P , Johnson WE, Roos C, Seuánez HN, Horvath JE, Moreira MAM, Kessing B, Pontius J, Roelke M, Rumpler Y, et al 2011. A molecular phylogeny of living primates. PLoS Genet. 7(3):e1001342. 21436896 10.1371/journal.pgen.1001342 PMC3060065

[msab022-B38] Pond SLK , Frost SDW. 2005. Datamonkey: rapid detection of selective pressure on individual sites of codon alignments. Bioinformatics 21(10):2531–2533. 15713735 10.1093/bioinformatics/bti320

[msab022-B39] Rogers SL , Kaufman J. 2016. Location, location, location: the evolutionary history of CD1 genes and the NKR-P1/ligand systems. Immunogenetics 68(8):499–513. 27457887 10.1007/s00251-016-0938-6 PMC5002281

[msab022-B40] Salomonsen J , Sørensen MR, Marston DA, Rogers SL, Collen T, van Hateren A, Smith AL, Richard K, Beal K, Skjødt J, Kaufman ND. 2005. Two CD1 genes map to the chicken MHC, indicating that CD1 genes are ancient and likely to have been present in the primordial MHC. Proc Natl Acad Sci U S A . 102:8668–8673. 15939887 10.1073/pnas.0409213102 PMC1150808

[msab022-B41] Sawyer SL , Wu LI, Emerman M, Malik HS. 2005. Positive selection of primate TRIM5αidentifies a critical species-specific retroviral restriction domain. Proc Natl Acad Sci U S A. 102:2832–2837. 15689398 10.1073/pnas.0409853102 PMC549489

[msab022-B42] Schito M , Migliori GB, Fletcher HA, McNerney R, Centis R, D’Ambrosio L, Bates M, Kibiki G, Kapata N, Corrah T, et al 2015. Perspectives on advances in tuberculosis diagnostics, drugs, and vaccines. Clin Infect Dis. 61(Suppl 3):S102–S118. 10.1093/cid/civ609 PMC4583570 26409271

[msab022-B43] Takahashi T , Suzuki T. 2012. Role of sulfatide in normal and pathological cells and tissues. J Lipid Res. 53(8):1437–1450. 22619219 10.1194/jlr.R026682 PMC3540844

[msab022-B44] Takahata N , Nei M. 1990. Allelic genealogy under overdominant and frequency-dependent selection and polymorphism of major histocompatibility complex loci. Genetics 124(4):967–978. 2323559 10.1093/genetics/124.4.967 PMC1203987

[msab022-B45] Thierry-Mieg D , Thierry-Mieg J. 2006. AceView: a comprehensive CDNA-supported gene and transcripts annotation. Genome Biol. 7(Suppl 1):S12. 16925834 10.1186/gb-2006-7-s1-s12 PMC1810549

[msab022-B46] Trott O , Olson AJ. 2009. AutoDock Vina: improving the speed and accuracy of docking with a new scoring function, efficient optimization and multithreading. J Comput Chem. 31(2):61. 10.1002/jcc.21334 PMC3041641 19499576

[msab022-B47] Van Valen L. 1973. A new evolutionary law. Evol. Theory 1: 1-30.

[msab022-B48] Yang J , Zhang Y. 2015. Protein structure and function prediction using I-TASSER. Curr Protoc Bioinformatics 52(December):5.8.1–5.815. 26678386 10.1002/0471250953.bi0508s52 PMC4871818

[msab022-B49] Yang Z. 2007. PAML 4: phylogenetic analysis by maximum likelihood. Mol Biol Evol. 24(8):1586–1591. 17483113 10.1093/molbev/msm088

[msab022-B50] Zajonc DM , Crispin MDM, Bowden TA, Young DC, Cheng T-Y, Hu J, Costello CE, Rudd PM, Dwek RA, Miller MJ, et al 2005. Molecular mechanism of lipopeptide presentation by CD1a. Immunity 22(2):209–219. 15723809 10.1016/j.immuni.2004.12.009

[msab022-B51] Zajonc DM , Elsliger MA, Teyton L, Wilson IA. 2003. Crystal structure of CD1a in complex with a sulfatide self antigen at a resolution of 2.15 A. Nat Immunol. 4(8):808–815. 12833155 10.1038/ni948

[msab022-B52] Zajonc DM , Flajnik MF. 2016. CD1, MR1, NKT, and MAIT: evolution and origins of non-peptidic antigen recognition by T lymphocytes. Immunogenetics 68(8):489–490. 27480412 10.1007/s00251-016-0941-y PMC5450827

