# Supplementary material for: Diversification of CD1 Molecules Shapes Lipid Antigen Selectivity
Source: Mol Biol Evol. 2021 Feb 2;38(6):2273–84. doi: 10.1093/molbev/msab022 (PMC8136489; doi:10.1093/molbev/msab022)
Supplement: msab022_Supplementary_Data [file msab022_supplementary_data.zip › MBE_CD1_SuppData.pdf]

Supplemental Data

Sequences used for phylogenetic analysis with NCBI accession identification numbers.

Primate CD1a, 18 species

Homo sapiens*,** NCBI Reference Sequence: NM_001763.3; ***Macaca mulatta*,** NCBI Reference Sequence: NM_001145818.1**;** ***Pan troglodytes*,** NCBI Reference Sequence: XM_001169121.4; ***Macaca fascicularis*,** NCBI Reference Sequence: XM_005595550.2; ***Papio anubis, NCBI Reference Sequence: XM_003892864.5; ***Gorilla gorilla gorilla,*** NCBI Reference Sequence: XM_004027022.3; ***Pan paniscus*,** NCBI Reference Sequence: XM_024926560.2**;** ***Pongo abelii,*** NCBI Reference Sequence: XM_002809984.3; ***Aotus nancymaae*,** NCBI Reference Sequence: XM_012449747.1; ***Rhinopithecus bieti,*** NCBI Reference Sequence: XM_017868910.1; ***Rhinopithecus roxellana*,** NCBI Reference Sequence: XM_030935243.1; ***Colobus angolensis palliatus*,** NCBI Reference Sequence: XM_011928485.1; ***Piliocolobus tephrosceles*,** NCBI Reference Sequence: XM_023214151.2 ***Cebus capucinus* imitator,** NCBI Reference Sequence: XM_017503703.1; ***Callithrix jacchus*,** NCBI Reference Sequence: XM_002764268.4; ***Chlorocebus sabaeus,*** NCBI Reference Sequence: XM_007976629.1; ***Cercocebus atys,*** NCBI Reference Sequence: XM_012093252.1; ***Macaca nemestrina,*** NCBI Reference Sequence: XM_011769959.2

Primate CD1b, 21 Species

Homo sapiens*,** GenBank: AK303330.1**;** ***Papio anubis, NCBI Reference Sequence: XM_003892865.5**;** ***Theropithecus gelada,*** NCBI Reference Sequence: XM_025362870.1**;** ***Mandrillus leucophaeus*** NCBI Reference Sequence: XM_011978334.1**;** ***Cercocebus atys*,** NCBI Reference Sequence: XM_012093255.1**;** ***Chlorocebus sabaeus*,** NCBI Reference Sequence: XM_007976624.1**;** ***Macaca fascicularis*,** NCBI Reference Sequence: XM_005541341.2**;** ***Macaca nemestrina,*** NCBI Reference Sequence: XM_011769969.2 ***Rhinopithecus roxellana*,** NCBI Reference Sequence: XM_010387232.2**;** ***Colobus angolensis palliatus,*** NCBI Reference Sequence: XM_011957139.1**;** ***Piliocolobus tephrosceles,*** NCBI Reference Sequence: XM_023214158.2**;** ***Nomascus leucogenys*,** NCBI Reference Sequence: XM_003258651.4; ***Pan troglodytes*,** NCBI Reference Sequence: XM_513909.5; ***Pongo abelii,*** NCBI Reference Sequence: XM_002809978.3; ***Gorilla gorilla gorilla,*** NCBI Reference Sequence: XM_004027024.3; ***Pan paniscus*,** NCBI Reference Sequence: XM_003821008.2; ***Cebus capucinus imitator,*** NCBI Reference Sequence: XM_017503705.1; ***Saimiri boliviensis boliviensis*,** NCBI Reference Sequence: XM_003937886.2; ***Aotus nancymaae*,** GenBank: AY605931.1; ***Callithrix jacchus,*** NCBI Reference Sequence: XM_002760142.3

Primate CD1c, 20 species

Homo sapiens, Reference Sequence: NM_001765.3; ***Papio anubis*,** NCBI Reference Sequence: XM_021925284.2; ***Theropithecus gelada,*** NCBI Reference Sequence: XM_025393420.1; ***Mandrillus leucophaeus,*** NCBI Reference Sequence: XM_011969349.1; ***Macaca mulatta*,** NCBI Reference Sequence: NM_001145533.1; ***Macaca fascicularis,*** NCBI Reference Sequence: XM_005595546.2, ***Macaca nemestrina,*** NCBI Reference Sequence: XM_011769962.2, ***Rhinopithecus bieti,*** NCBI Reference Sequence: XM_017868926.1**;** ***Rhinopithecus roxellana,*** NCBI Reference Sequence: XM_010381246.2; ***Piliocolobus tephrosceles,*** *NCBI* Reference Sequence: XM_023214153.2; ***Colobus angolensis palliates,*** NCBI Reference Sequence: XM_011928487.1; ***Nomascus leucogenys,*** *NCBI* Reference Sequence: XM_003258650.2; ***Pongo abelii,*** NCBI Reference Sequence: XM_002809979.3; ***Pan paniscus,*** NCBI Reference Sequence: XM_003821009.3**;** ***Gorilla gorilla gorilla**,* NCBI Reference Sequence: XM_019025012.2; ***Pan troglodytes,*** NCBI Reference Sequence: XM_513908.6; ***Cebus capucinus imitator,*** NCBI Reference Sequence: XM_017503701.1; ***Cercocebus atys,*** NCBI Reference Sequence: XM_012093253.1; ***Callithrix jacchus*,** NCBI Reference Sequence: XM_035279155.1

Primate CD1d, 18 species

Homo sapiens, NCBI Reference Sequence: NM_001766.4; ***Aotus nancymaae,*** NCBI Reference Sequence: XM_012449750.2; ***Rhinopithecus roxellana,*** NCBI Reference Sequence: XM_030935246.1; ***Cercocebus atys,*** NCBI Reference Sequence: XM_012093246.1; ***Papio anubis,*** NCBI Reference Sequence: XM_017948205.3; ***Macaca fascicularis,*** NCBI Reference Sequence: XM_005541342.2; ***Macaca nemestrina,*** NCBI Reference Sequence: XM_011769953.1; ***Chlorocebus sabaeus,*** NCBI Reference Sequence: XM_007976632.1; ***Pongo abelii,***

NCBI Reference Sequence: XM_024247050.1; ***Pan troglodytes,*** NCBI Reference Sequence: NM_001071804.1; ***Pan paniscus,*** NCBI Reference Sequence: XM_008974483.3 ***Macaca mulatta,*** NCBI Reference Sequence: NM_001033114.2; ***Gorilla gorilla gorilla,*** NCBI Reference Sequence: XM_019024988.2; ***Aotus nancymaae,*** NCBI Reference Sequence: XM_012449750.2; ***Saimiri boliviensis boliviensis,*** NCBI Reference Sequence: XM_010348509.1; ***Piliocolobus tephrosceles,*** NCBI Reference Sequence: XM_023214147.2; ***Rhinopithecus bieti,*** NCBI Reference Sequence: XM_017868929.1; ***Colobus angolensis palliates,*** NCBI Reference Sequence: XM_011928483.1;

Primate CD1e, 20 species

Homo sapiens, NCBI Reference Sequence: NM_030893.4; ***Rhinopithecus roxellana*****,** NCBI Reference Sequence: XM_030935233.1; ***Macaca nemestrina,*** NCBI Reference Sequence: XM_011769970.2; ***Macaca fascicularis,*** NCBI Reference Sequence: XM_015455159.1*;* ***Cercocebus atys*****,** NCBI Reference Sequence: XM_012093256.1; ***Chlorocebus sabaeus,*** NCBI Reference Sequence: XM_007976621.1; ***Theropithecus gelada,*** NCBI Reference Sequence: XM_025356228.1; ***Aotus nancymaae*****,** NCBI Reference Sequence: XM_012449741.1; ***Pongo abelii,*** NCBI Reference Sequence: XM_003775532.3; ***Saimiri boliviensis boliviensis,*** NCBI Reference Sequence: XM_003937882.2; ***Cercocebus atys,*** NCBI Reference Sequence: XM_012093259.1; ***Gorilla gorilla gorilla,*** NCBI Reference Sequence: XM_004027025.3; ***Pan troglodytes,*** NCBI Reference Sequence: XM_513910.6; ***Pan paniscus,*** NCBI Reference Sequence: XM_003821003.4; ***Nomascus leucogenys*****,** NCBI Reference Sequence: XM_003258652.4; ***Rhinopithecus bieti,*** NCBI Reference Sequence: XM_017868912.1; ***Piliocolobus tephrosceles,*** NCBI Reference Sequence: XM_023214159.1; ***Colobus angolensis palliatus,*** NCBI Reference Sequence: XM_011957140.1; ***Papio anubis,*** NCBI Reference Sequence: XM_021925303.2; ***Mandrillus leucophaeus*****,** NCBI Reference Sequence: XM_011978335.1;
