# Supplementary material for: Diversification of CD1 Molecules Shapes Lipid Antigen Selectivity
Source: Mol Biol Evol. 2021 Feb 2;38(6):2273–84. doi: 10.1093/molbev/msab022 (PMC8136489; doi:10.1093/molbev/msab022)
Supplement: msab022_Supplementary_Data [file msab022_supplementary_data.zip › MBE_CD1_SuppFigs.pdf]

Supplemental Figures 1-8

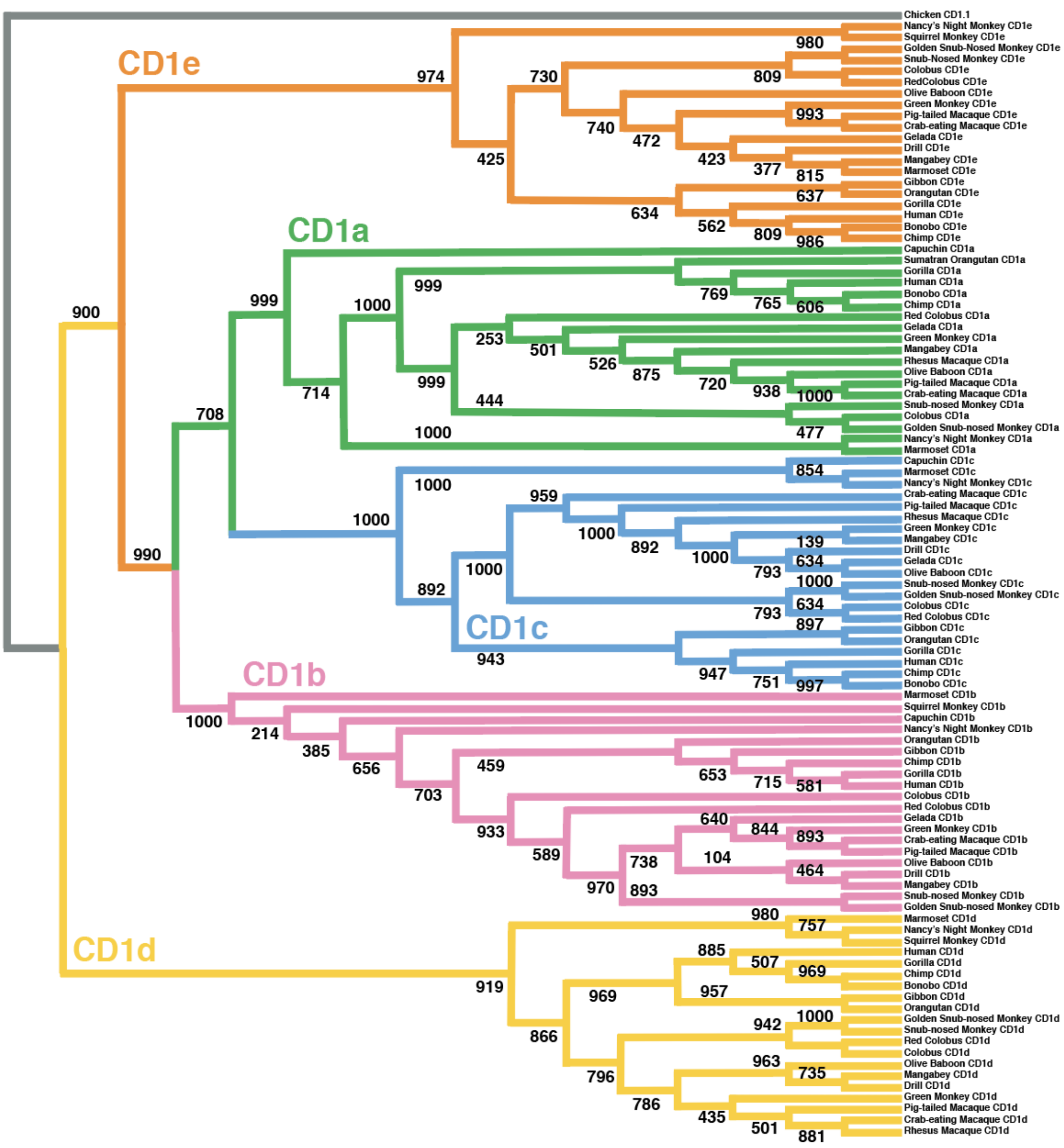

Figure S1. CD1 Primate Family Tree

Phylogenetic relationship of primate CD1 homologs used in this study. Tree was generated in PhyML with 1000 bootstraps. Chicken CD1.1 was included as an outgroup.

Species	Bounding box x	Bounding box y	Bounding box z	Flexible residues
Human	size_x = 82	size_y = 114	size_z = 126	F143-M157
Chimpanzee	size_x = 124	size_y = 76	size_z = 114	V141-N156
Bonobo	size_x = 126	size_y = 98	size_z = 124	L138-M146
Orangutan	size_x = 110	size_y = 90	size_z = 126	P150-N156
Gorilla	size_x = 118	size_y = 126	size_z = 92	L149-N156
Olive Baboon	size_x = 78	size_y = 98	size_z = 122	L149-N156
Green Monkey	size_x = 118	size_y = 82	size_z = 126	L149-N156
Mangabey	size_x = 126	size_y = 110	size_z = 108	P150-N156
Pig-Tailed Macaque	size_x = 110	size_y = 126	size_z = 90	D72-N91
Crab-eating Macaque	size_x = 84	size_y = 124	size_z = 126	T71-N92
SnubNoseMonkey	size_x = 100	size_y = 96	size_z = 126	L57-N77 E82 L83 L86 I174 L178
GoldenSnubNoseMonkey	size_x = 124	size_y = 108	size_z = 116	A150-N156 L86 I174
Colobus	size_x = 82	size_y = 118	size_z = 126	L57-N77
Marmoset	size_x = 78	size_y = 108	size_z = 108	F66-N77 L83 V97 L161 L178 V174
Capuchin	size_x = 100	size_y = 124	size_z = 96	F143-N156 L86 V170 V173 L174 R177

Figure S2. Vina Settings: Grid box parameters and flexible residues

Parameter settings for config file used to generate ligand docking models with Autodock Vina, including residues set as flexible which correspond to the loop residues between alpha helices 1 and 2, and any occluding residues not engaged in hydrogen bonding at the portal entrance.

-	CD1a	CD1b	CD1c	CD1d	CD1e
Co-receptor	αβ TCR	γδ TCR	iNKT TCR	TypeII NKT TCR	MAIT cell TCR
Mycobacterial ligand	Didehydroxymycobactin	Mycolic acids (Mycobacterium)	Mannosyl-β1-phosphomycoketides (Mycobacterium)	Phosphatidylinositol mannosides	Phosphatidylinositol mannosides (indirect)
Ligand in crystal structure	Lysophosphatidylcholine (self)	GM2 ganglioside (self)	Phosphomycoketide (Mycobacterium)	Sulfatide (self)	-
-	Sphingomyelin (self)	Phophatidylinositol (self)	Phosphatidylcholine (self)	Lysophosphatidylcholine (self)	-
-	Sulfatide (self)	Phosphatidylcholine (self)	Mannosyl-β1-phosphomycoketide (Mycobacterium)	Sphingomyelin (self)	-
-	Synthetic mycobactin lipopeptide	Phosphatidylserine (self)	-	Glycosphingolipid (self)	-
-	-	Phosphatidic acid (self)	-	Ganglioside GD3 (self)	-
-	-	Glucose monomycolate, C36 GMM 9 (Mycobacterium)	-	-	-

Figure S3. CD1 family members recognize a variety of mycobacteria-derived and endogenous lipids

Multiple mycobacterial lipids and lipoproteins are recognized by CD1 receptors, suggesting that this bacterial family known for exotic lipids has been interacting with the CD1 receptors across an extended timespan.

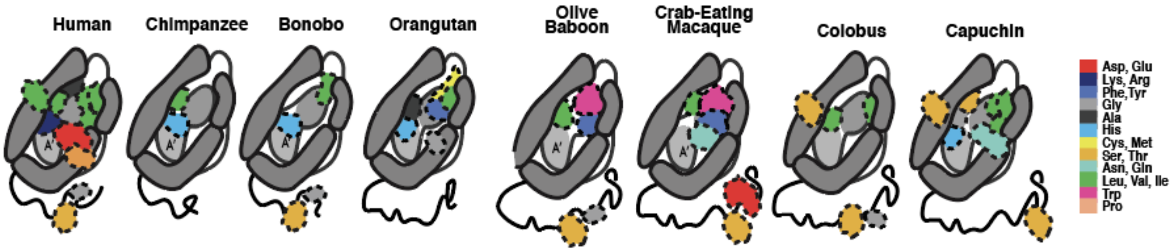

Figure S4. Sites under positive selection that differ from consensus, illustrated in a visual cartoon. Property differences in amino acids cluster around the portal and at the TCR interaction surface, while internal residues are mainly hydrophobic residues of varying sizes.

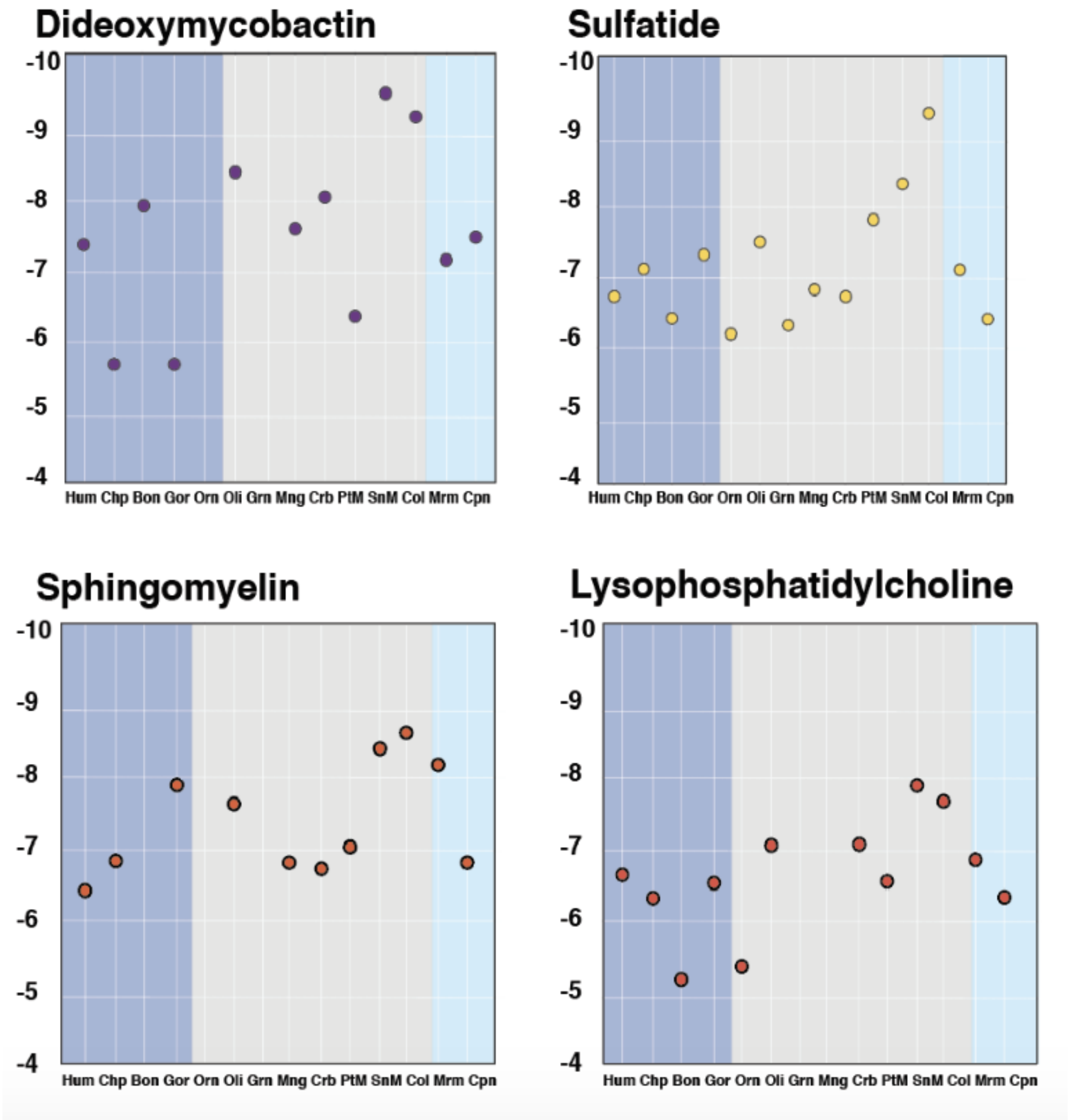

Figure S5. Plots of CD1a primate docking experiments by individual lipid.

Dark blue-Hominoids, **Grey**-Old World Monkeys, **Light blue**-New World Monkeys.

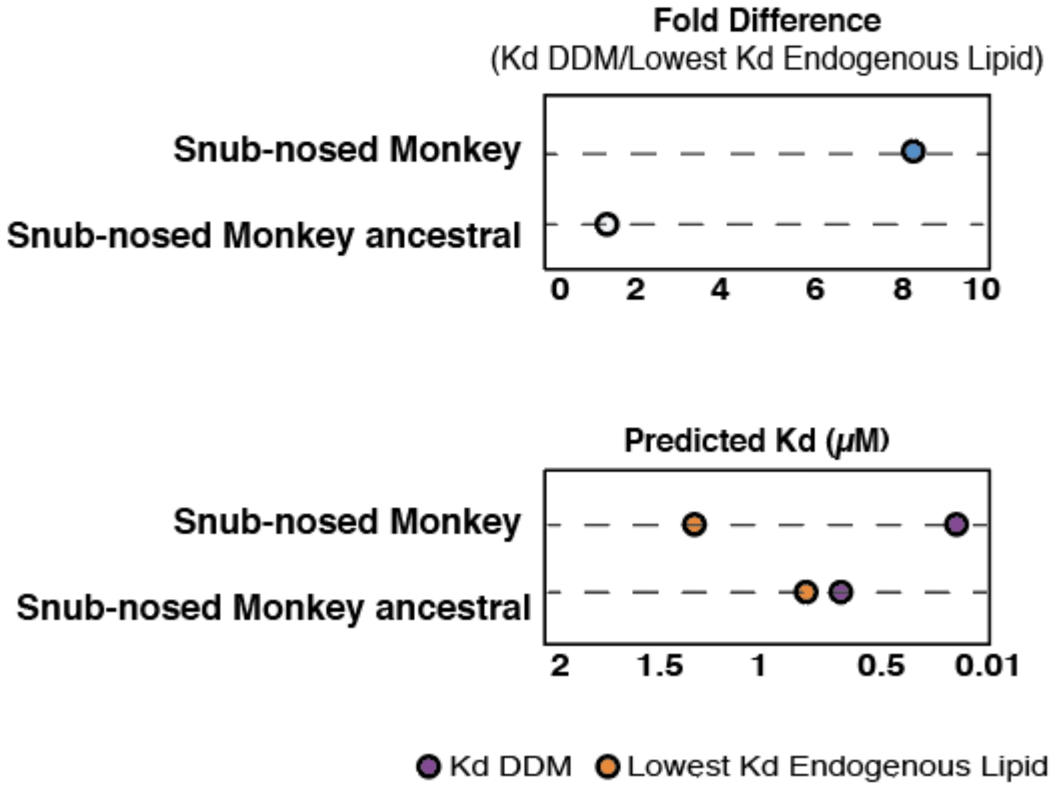

Figure S6. Snub-nosed monkey represents consensus at sites under selection. Ancestrally-predicted amino acids differ from consensus. Reversion of sites to ancestral state in snub-nosed monkey background results in lower affinity for DDM and higher affinity for endogenous lipid.

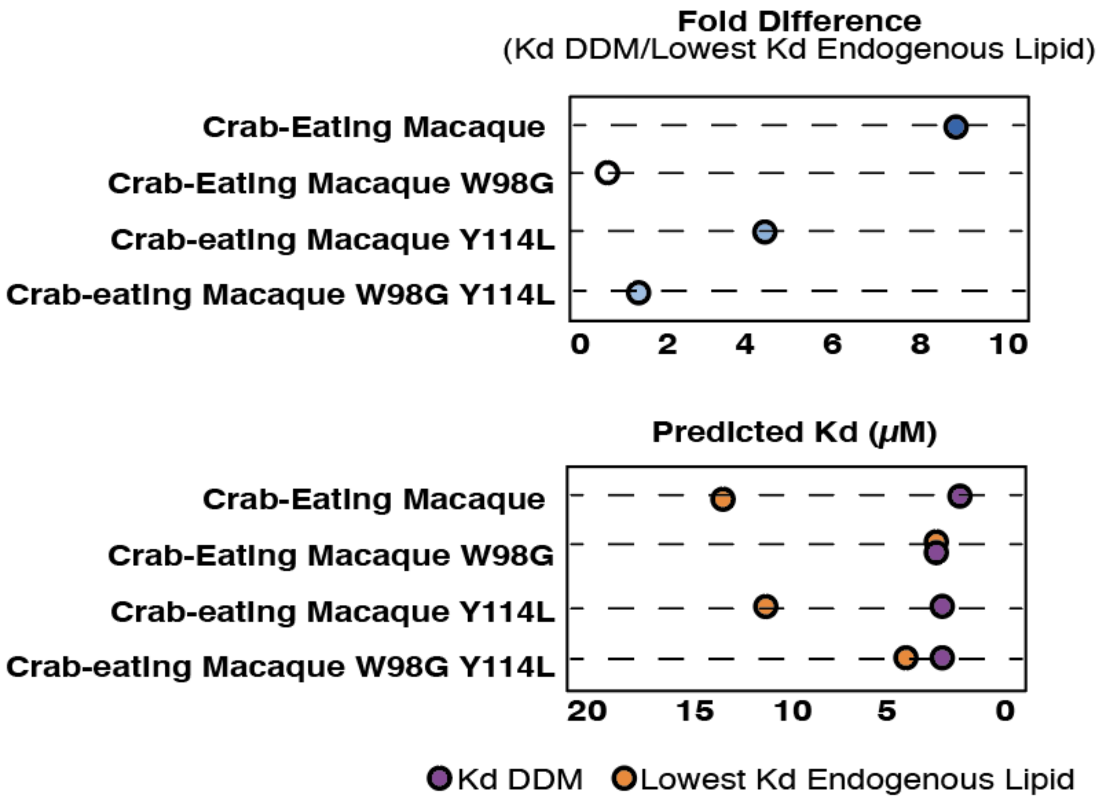

Figure S7. Additional amino acid changes confer minor increase in spread between endogenous and exogenous lipid. Small changes in affinity can be seen when other sites with high omega values are plotted in the Crab-eating macaque, such as site 114 which appears to have a similar effect to site 98, though smaller in magnitude, presumably due to the loss of bulky residue in the deeper chambers of the pocket.

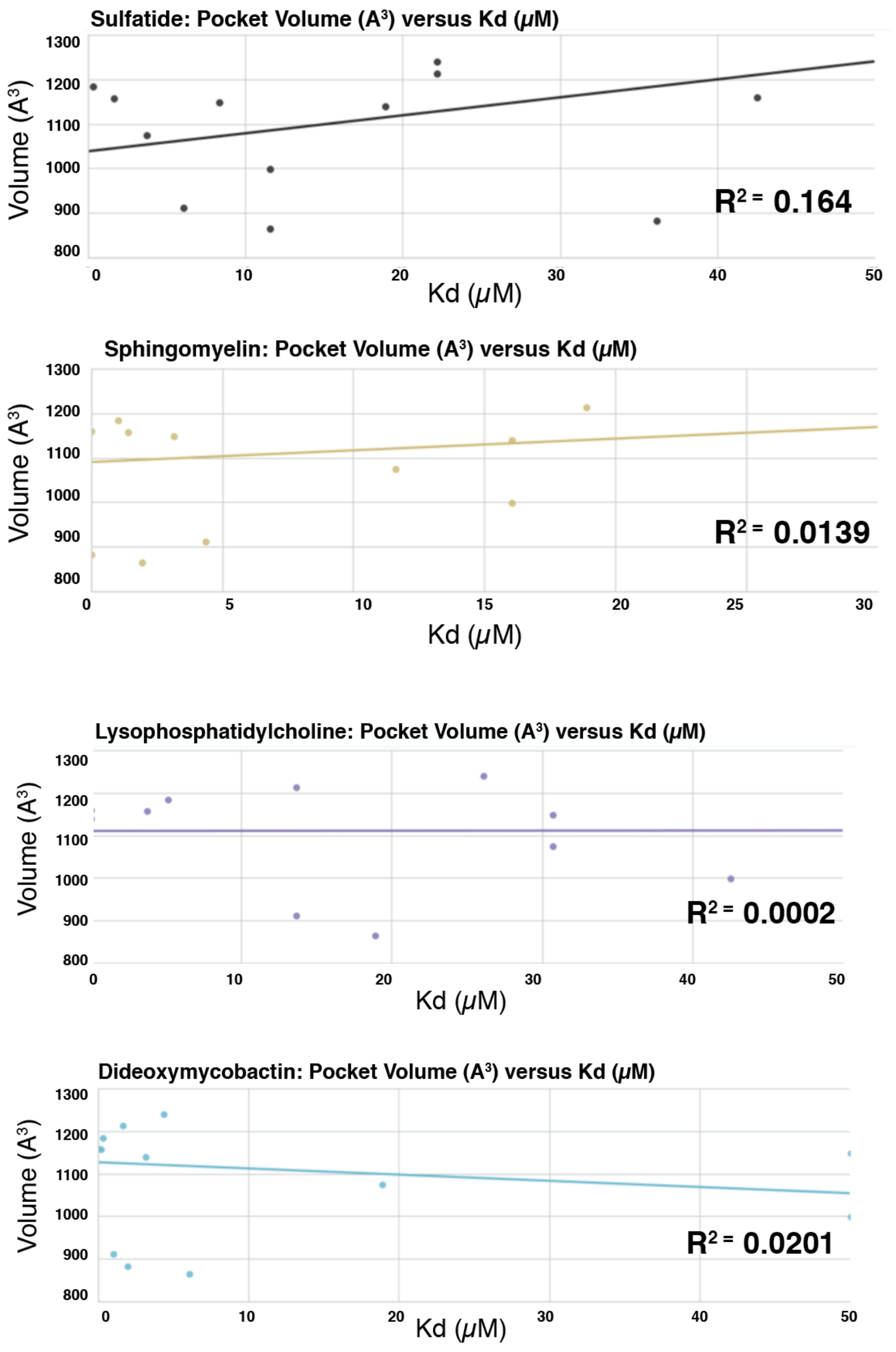

Figure S8. Pocket volume does not correlate with lipid binding affinity. Plots of docking results were converted to Kd (dissociation constant) and plotted against predicted pocket volumes. Observations did not support a case where larger pocket was associated with better binding.
